# Curcumin 3.0—Therapeutic and Diagnostic Potential in Cancer and Beyond

**DOI:** 10.3390/ijms23105398

**Published:** 2022-05-12

**Authors:** Beatrice E. Bachmeier, Roman Blaheta

**Affiliations:** 1 Institute of Pharmaceutical Biology, Goethe University Frankfurt, 60438 Frankfurt am Main, Germany; 2 Department of Urology and Pediatric Urology, University Medicine Mainz, 55131 Mainz, Germany; roman.blaheta@unimedizin-mainz.de

Curcumin is one of the most interesting plant-derived polyphenols with a high potential for therapeutic, and even diagnostic, application in various diseases. Since the start of the millennium, the number of publications addressing its (pre-)clinical research results and benefits has been continuously rising, ranging from 108 Pubmed-listed records in the year 2000 to 2379 in 2021.

This Special Issue, which is already on its third volume, addresses novel research findings about this outstanding compound. Nine papers have been published, including five original articles and four reviews, focussing on the pharmacological effects of Curcumin.

Despite Curcumin’s therapeutic potential in vitro and in vivo, there is an ongoing discussion about the lipophilic character of this molecule. At low concentrations, curcumin is water-soluble; however, it has not been explored in depth if the concentrations that can realistically be reached in the blood stream are high enough to exert pharmacological effects. In fact, it is not fully understood how Curcumin is transported to the target sites in order to exert its therapeutic effects. Still, in vivo studies have already documented the anti-metastatic effects of orally administered Curcumin [1,2].

To overcome pharmacological limitations, particularly caused by low absorption and rapid metabolism, many efforts have been undertaken to enhance the **bioavailability** of Curcumin. This includes the encapsulation of Curcumin into nanoparticles, the application of Curcumin in combination with visible light or the development of Curcumin derivatives. All these endeavours have been undertaken to exploit Curcumin’s therapeutic potential as an **anti-neoplastic, neuroprotective or anti-microbial drug**. In the current Special Issue, three groups present their original data on this eagerly discussed research objective. The original papers are complimented by two interesting review articles addressing the problem of bioavailability:

- The research article by Saeed et. al. discloses the binding affinity of 50 Curcumin derivatives to known cancer-related targets, among them, epidermal growth factor receptor (EGFR) and nuclear factor κB (NF-κB) [3]. Curcumin has been described as a potent inhibitor of NF-κB activation, a key process in tumour progression and metastasis, as well as in inflammatory processes. EGFR plays a central role in the pathogenesis and progression of different carcinoma types and is overexpressed in many human carcinomas. EGFR is also involved in developing resistance to chemotherapy. By using a molecular docking approach, 20 out of 50 Curcumin derivatives showed binding energies to NF-κB smaller than −10 kcal/mol, while Curcumin as a lead compound revealed free binding energies of >−10 kcal/mol. Comparable data were obtained for EGFR: 15 out of 50 Curcumin compounds were bound to EGFR with free binding energies of <−10 kcal/mol, while the binding affinity of Curcumin itself was >−10 kcal/mol. This indicates that the derivatization of Curcumin may indeed be a promising strategy to improve target specificity and to obtain more effective anticancer drug candidates. Additionally, these research results reveal that molecular docking represents a valuable approach to facilitate and speed up the identification of novel targeted Curcumin-based drugs to treat cancer.
- Dokovic and coworkers developed novel PEGylated nanoemulsions as parental delivery system for Curcumin for parenteral application [4]. Curcumin encapsulated in these nanoparticles remained stable and maintained its antioxidant capacity and resided in the plasma up to 20 min after intravenous administration. The nanoparticles have been tested in vitro for cytotoxicity and found to be safe; however, thorough in vivo testing has to be performed before such formulations can be clinically applied.
- The Review by Prasad and coworkers summarizes the efforts that have been undertaken so far to develop therapeutic and diagnostic tools based on complexes of metals with Curcumin [5]. In contrast to the lipophilic Curcumin molecules, metal–Curcumin complexes have an increased solubility, cellular uptake and bioavailability. Despite the promising results that have been observed in preclinical studies ranging from antioxidant and anti-inflammatory to antimicrobial and antiviral effects, metal complexes are highly cytotoxic and should be applied with great care and caution. The advantages should be carefully weighed against their disadvantages in clinical application, and preclinical results from in vitro studies should be interpreted with responsibility and diligence.
- The Review by Trigo-Gutierrez and colleagues summarizes the research efforts of encapsulating Curcumin in drug delivery systems and the antimicrobial properties of these new compounds [6]. This carefully composed review gives an excellent overview of the various methods and techniques of nanocarriers and weighs diligently between therapeutic advantages and possible risks of clinical application. The antimicrobial activity of encapsulated Curcumin against viruses, bacteria and fungi, including resistant and emergent pathogens, is excellently delineated. Moreover, the authors explain that most of the studies with encapsulated Curcumin have been performed in vitro, and the lack of clinical studies accounts for the poor transfer of preclinical knowledge in clinical application. However, this is well justified because the safety of novel therapeutic applications always has priority.
- The original research article of Rutz and colleagues describes their interesting approach to optimize the pharmacological efficacy of Curcumin by the application of visible light irradiation [7]. The researchers found that tumour growth and metastasis are reduced when prostate cancer cells are treated with low-dose Curcumin and subsequent irradiation with visible light. Deciphering the underlying cell biological mechanisms, they show that the combination therapy inhibited tumour cell adhesion, migration, phosphorylation of CDK1 and the expression of their receptors Cyclin A and B. In line with diminished adhesion, the expression of integrin subtypes α and β were also reduced. These results are in concordance with previous findings by the group [8] and emphasize the potential of applying light irradiation to enhance the anti-tumour potential of Curcumin. Additionally, the combination of Curcumin and visible light irradiation is a smart therapeutic application to overcome Curcumin’s pharmacological limitations without introducing toxicity.

Generally, the anti-tumorigenic effect of Curcumin is an extremely interesting research topic. In this Special Issue, the influence of Curumin on two **rare tumour types**, glioblastoma and mesothelioma, has also been dealt with:

- Glioblastoma is an extremely aggressive and hard to treat brain tumour. Despite treatment by radiation and chemotherapy after surgical elimination of the tumour, patients have an extremely bad prognosis, with survival rates of less than one year. Majchrzak-Celinska and coworkers describe the effects of Curcumin combined with the natural histone deacetylase inhibitor sodium butyrate [9]. The relevance of this regimen to treat glioblastoma has not been explored so far. Delineating the molecular mechanism, the researchers detected a decrease in glioblastoma cells’ viability, synergistically driven by the two compounds. Curcumin and sodium butyrate administered as single compound or in combination induced apoptosis and cell cycle arrest, although to a different degree depending on the particular glioblastoma cell line used. Apoptosis induction and cell cycle arrest correlated with distinct alterations of cell-growth-related gene expression. Although these promising preclinical studies point to beneficial effects of the drug combination in vitro, there is still a long way to clinical application in particular, as cytotoxic effects cannot be excluded.
- Mesothelioma is a rare and aggressively growing form of cancer. Several mesothelioma treatment options have been established, but they are palliative rather than curative. In this context, Pouliquen and coworkers have examined the effects of intraperitoneal administration of curcumin in vivo [10]. The results of the study indicate that this protocol targets secondary lymphoid organs of tumor-bearing rats at two complementary levels—in spleen and in lymph nodes—leading to a restored immune defense. Furthermore, Curcumin modulated the expression of a series of biomarkers in lymphoid organs, indicating an out-of-field systemic antitumor effect distant to the tumor site, probably related to the massive induction of necrosis/apoptosis in tumor cells. These observations provide an intriguing basis for future mechanistic studies.

In addition to anti-tumorigenic and antimicrobial effects, the review by Nebrisi addresses Curcumin’s **neuroprotective potential** [11]. Parkinson’s disease is the second most common neurodegenerative disease after Alzheimer’s disease and is a slowly progressive multisystem disorder rather than just a disease, involving massive neuropathological degeneration in dopaminergic neurons. In this context, this review focuses on the neuroprotective activities of curcumin in Parkinson’s disease and the various mechanisms involved. Information about Curcumin’s pharmacokinetics, pharmacodynamics, biological, cellular, and molecular properties are provided. A special emphasis is given to Curcumin’s neuroprotective activities via an α7-nAChR-mediated mechanism, on its safety profile and on current and upcoming clinical trials for clinical application. The review supports Curcumin’s powerful molecular and cellular effects in neurodegenerative disorders as an appealing strategy for improving Parkinson’s disease management.

Last but not least, the review by Mari and colleagues summarizes the results of **radiolabelled Curcumin as a radiotracer** [12]. The development of curcumin-based radiotracers represents a useful approach not only to elucidate metabolic and pharmacokinetics aspects of promising molecules but also to obtain suitable pharmaceuticals for imaging purposes in nuclear medicine applications. Nuclear medicine is a branch of medicine that utilizes such radioactive compounds for diagnoses or therapies of diseases. Synthetic pathways, labelling methods and the preclinical investigations involving these radioactive compounds are summarized, including chemical modifications for enhancing Curcumin’s stability, as well as its functionalization for the labelling with several radio-halogens or metal radionuclides. So far, several radiolabeled Curcumin and curcuminoid derivatives have been studied with the main goals of providing sensible radiotracers for the early diagnosis of Alzheimer’s disease and for the detection of neoplastic lesions by means of nuclear medicine instrumentations. Although almost all the radiolabeled derivatives exhibited good affinity for Aβ-amyloid plaques in vitro, none of them proved to be a suitable tool for the imaging of Alzheimer’s disease in vivo, probably since the modification to the curcumin backbone hinder trafficking through the blood–brain barrier. Concerning the application as radiotracers for the diagnosis of tumors, the curcumin derivatives tested in vivo so far were clearly able to visualize the lesion but showed a widespread distribution in many organs. This finding hampers the direct use of radiolabeled Curcumin derivatives as diagnostic tools, but the studies performed hitherto lay the foundation for developing further curcumin-based compounds with improved characteristics.

In conclusion, this Special Issue highlights novel insights into molecular mechanisms underlying the diagnostic and therapeutic benefits of Curcumin and its derivatives for many applications. While a lot of research has been conducted so far, especially in deciphering the molecular and cellular pathways and chemical characteristics of the original compound and its derivatives, the missing in vivo and clinical studies on toxicity and efficacy often make it difficult to pave the way for therapeutic applications.
